# Variations in tetrodotoxin levels in populations of *Taricha granulosa* are expressed in the morphology of their cutaneous glands

**DOI:** 10.1038/s41598-019-54765-z

**Published:** 2019-12-06

**Authors:** Pedro Luiz Mailho-Fontana, Carlos Jared, Marta Maria Antoniazzi, Juliana Mozer Sciani, Daniel Carvalho Pimenta, Amber N. Stokes, Taran Grant, Edmund D. Brodie, Edmund D. Brodie

**Affiliations:** 1 Instituto Butantan, São Paulo, Brazil; 2 Universidade São Francisco, Bragança Paulista, Brazil; 3 California State University, Bakersfield, USA; 4 University of São Paulo, São Paulo, Brazil; 5 University of Virginia, Charlottesville, USA; 6 Utah State University, Logan, USA

**Keywords:** Biodiversity, Herpetology

## Abstract

Tetrodotoxin (TTX), one of the most toxic substances in nature, is present in bacteria, invertebrates, fishes, and amphibians. Marine organisms seem to bioaccumulate TTX from their food or acquire it from symbiotic bacteria, but its origin in amphibians is unclear. *Taricha granulosa* can exhibit high TTX levels, presumably concentrated in skin poison glands, acting as an agent of selection upon predatory garter snakes (*Thamnophis*). This co-evolutionary arms race induces variation in *T. granulosa* TTX levels, from very high to undetectable. Using morphology and biochemistry, we investigated differences in toxin localization and quality between two populations at the extremes of toxicity. TTX concentration within poison glands is related to the volume of a single cell type in which TTX occurs exclusively in distinctive secretory granules, suggesting a relationship between granule structure and chemical composition. TTX was detected in mucous glands in both populations, contradicting the general understanding that these glands do not secrete defensive chemicals and expanding currently held interpretations of amphibian skin gland functionality. Skin secretions of the two populations differed in low-mass molecules and proteins. Our results demonstrate that interpopulation variation in TTX levels is related to poison gland morphology.

## Introduction

Tetrodotoxin (TTX) is one of the most toxic and well-studied but still mysterious natural products. TTX selectively binds to voltage-gated sodium channels in muscle and nerve tissues, causing paralysis and death^1^. It was first isolated from pufferfish (Tetraodontidae) but is now known to be distributed across a vast diversity of organisms. Among eukaryotes, it has been identified in distantly related invertebrates, including dinoflagellates, flatworms, bivalves, gastropods, sea slugs, crabs, starfish, and octopuses, and vertebrates, where it is restricted to amphibians and fishes^1–3^.

The rough-skin newt, *Taricha granulosa* (Salamandridae), possesses TTX in the skin, which it employs in chemical defense^4–10^. The amount of TTX present in the skin of individual *T. granulosa* varies extensively across populations, from undetectable to levels sufficient to kill 25,000 mice^6^. This extreme variation is driven by an evolutionary arms race with TTX-resistant garter snakes (*Thamnophis* spp.; Colubridae)^11–13^.

Despite decades of studying the chemistry, pharmacology, ecology, and evolution of TTX, its biogenesis remains poorly understood^14^. In marine organisms, the most widely accepted hypothesis is that TTX is produced by symbiotic bacteria and bioaccumulation^14,15^, but this model does not seem to apply to at least some of the amphibians that possess TTX. Although studies of captive-reared specimens of the frog *Atelopus varius*^16^ and the newt *Cynops pyrrhogaster*^17^ were found to lack or lose TTX over time, a study on *T. granulosa* reported increasing amounts of TTX in the skin over time in captive individuals^10^. Further, no TTX-producing bacteria have been isolated from any amphibian, and^18^ demonstrated that symbiotic bacteria are not present in or on newt skin, raising the possibility that newts might produce TTX themselves.

In addition to the lack of clarity regarding the origin of TTX in amphibians, little is known about the processes by which TTX is accumulated in the skin. Some studies found TTX to be concentrated in the granular glands of *Cynops pyrrhogaster*^19^ and *Notophthalmus viridescens*^20^, but to date no study has examined the skin of conspecific individuals with high and low levels of cutaneous TTX. In an effort to understand the process(es) involved in TTX accumulation in amphibians, we investigated the skin, especially the cutaneous glands, of *T. granulosa* by comparing the morphology and immunohistochemistry of individuals sampled from two populations, one with undetectable TTX levels and the other with high levels of TTX. We also examined the broader toxin profile of skin secretions of these populations using a biochemical approach.

## Results

### TTX quantification

Among the newts sampled randomly from the LW^−^ population, only one had detectable levels of TTX with a whole skin of a newt estimate of 0.0001 mg TTX (Table S1). In contrast, all newts from the SC^+^ population had much higher levels of TTX, with whole newt skin estimates varying between 1.05 mg and 1.38 mg TTX (Table S1).

### Morphology and histochemistry of cutaneous glands

**Figure 1:**
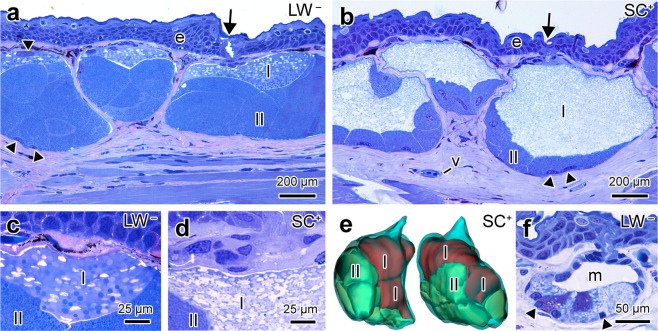
Characterization of skin morphology of *Taricha granulosa* from LW^−^ and SC^+^ populations. (**a**) Overview of skin from LW^−^ population showing the poison glands. Note that these glands are composed of two distinct types of secretory cells (I and II) completely filled with secretory granules. (**b**) Overview of skin from SC^+^ population, showing Type I cells within the voluminous poison glands. Note that in both populations Type I cells are always located immediately below the epithelial duct, through which the secretion is released (arrow). Note, also, that poison gland cells may exhibit more than one nucleus (arrowhead). (**c**) In the LW^−^ population, Type I cells contain two types of secretory granules, one spherical and dense, and the other elliptic and flocculent. (**d**) In the SC^+^ population, Type I cells are filled exclusively with flocculent elliptical granules. (**e**) Three-dimensional reconstruction of the poison gland of SC^+^ population, showing the relationship between Type I cells and the duct. (**f**) In the two populations, the mucous glands (m) have very similar characteristics and consist of two types of secretory cells that are identified by differences in staining. Note the rather obvious lumen and the presence of only one nucleus per cell. Epidermis (**e**), blood vessel (v). Staining: toluidine blue and fuchsine. Sexes of the animals: female (images **a**,**b**,**f**), male (images **c**–**e**).

*Taricha granulosa* skin presents many, evenly distributed poison glands (granular glands; Fig. 1a,b). Structurally, these glands are composed of several secretory cells that completely fill the interior of the gland and do not form a lumen (Fig. 1a–e). Each of the secretory cells possesses at least two peripherally arranged nuclei, and the cytoplasm is completely filled with secretory granules (Fig. 1a,b). No consistent differences were found in the morphology of male and female skin glands.

**Figure 2:**
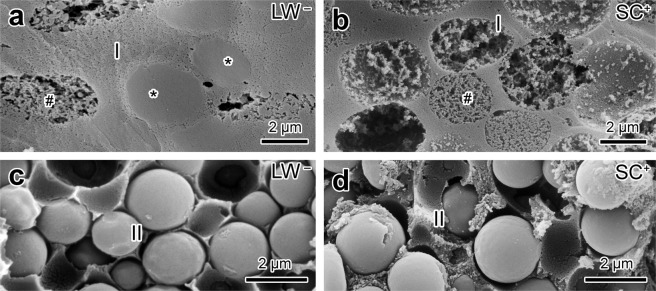
Scanning electron microscopy of the poison glands of *Taricha granulosa* from LW^−^ and SC^+^ populations. (**a**) In the LW^−^ population, Type I cells have two types of granules, a smaller one with a dense appearance (*) and a larger one with a flocculent appearance (#). (**b**) In the SC^+^ population, Type I cells exhibit only flocculated granules. (**c**) In the LW^−^ population, granules of Type II cells are spherical and show a smooth surface. (**d**) In the SC^+^ population, Type II cells are similar to those of the LW^−^ population. Sexes of the animals: female (images a–d).

The secretory cells comprising the poison glands can be classified into two types, Type I and Type II, according to their position within the gland and the morphological and histochemical characteristics of their secretory granules. Type I cells are located immediately below the duct (Fig. 1a–e). In the LW^−^ population, these cells generally present two kinds of granules, one rounded with a dense appearance and the other elliptic with a flocculent appearance (Figs. 1c and 2a). In the SC^+^ population, Type I cells are much larger than those of LW^−^ population and possess cytoplasm that is exclusively filled by flocculate granules (Figs. 1d and 2b). Histochemically, Type I cells did not show reactivity for any of the histochemical methods tested (Fig. S1). Type II cells are distributed along the sides and bottom of the poison gland, filling the glandular volume that is not occupied by Type I cells (Fig. 1a–e). In both populations, Type II cells show the same morphological characteristics and are filled with small, dense, spherical granules (Figs. 1a–d and 2c,d) with a protein-rich content (Fig. S1).

No significant difference was observed between the number or total volume of poison glands in the two populations (Table 1). However, the two populations differed significantly in the partial volumes occupied by Type I and II cells. The volume of Type I cells is much larger in the SC^+^ population than in the LW^−^ population (Table 1), whereas Type II cells occupy a much larger volume in the LW^−^ population than in the SC^+^ population (Table 1).

**Table 1:** Morphometric characterization of the poison glands of *Taricha granulosa* LW^−^ and SC^+^ populations.

Population	Sex of specimen	Number of glands per mm^2^	Number of cells Type I	Number of cells Type II	Poison gland vol (µL)	Σ volume Type I cells (%)	Σ volume Type II cells (%)
Lake in the Woods (LW^−^)	M F M M	18 14 13 16	1 1 3 1	11 10 7 8	0.00769 0.00761 0.00299 0.00347	16.83 18.00 39.68 5.80	83.17 82.00 60.32 94.20
Soap Creek (SC^+^)	M F F M	13 15 19 18	1 1 1 1	8 6 4 7	0.01147 0.00579 0.00380 0.00443	87.52 76.83 82.96 63.23	12.47 23.17 17.03 36.77
p value		0.5921	0.391	0.07	0.682	0.0006*	0.0006*

Note that the populations differed with respect to the relative volume of two types of cells within the poison glands. Significant statistical difference (*). Male (M), female (F).

Mucous glands exhibited similar morphology in both populations. These glands are constituted by two different mononucleate cell types that, in contrast to the poison glands, are arranged in a monolayer forming a well-defined central lumen that stores the secretion, which is subsequently released to the skin surface through an epithelial duct (Fig. 1f). One of the cell types is less abundant and exhibits granules that strongly and homogenously stain with toluidine blue, while the other cell type contains granules of different sizes and affinities to toluidine blue (Fig. 1f). Histochemically, most cells of the mucous glands do not react to any of the methods tested, and only some cells are positive for neutral mucopolysaccharides (Fig. S1).

### Immunohistochemistry

**Figure 3:**
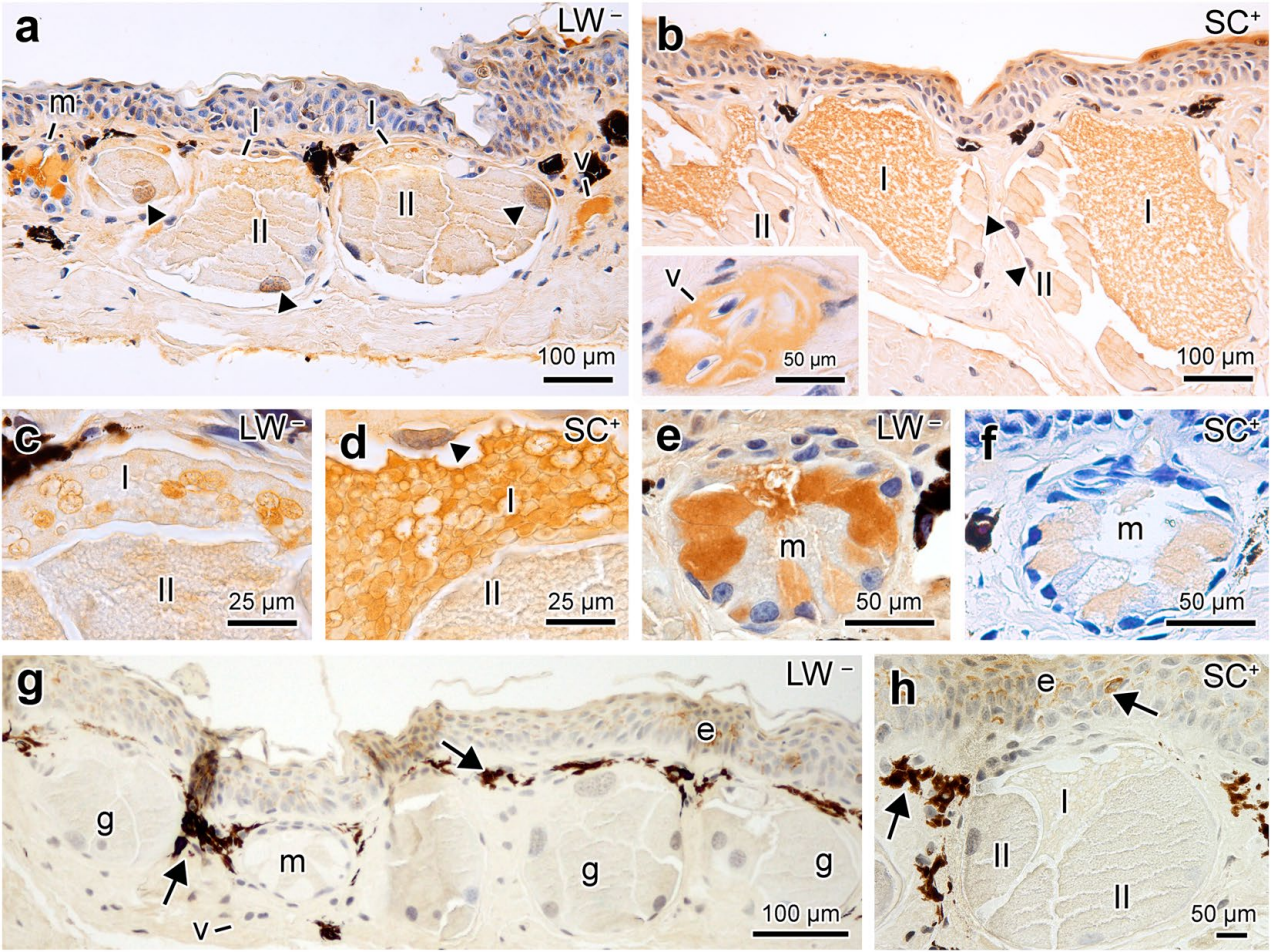
TTX immunolocalization in the skin of *Taricha granulosa* from LW^−^ and SC^+^ populations. (**a**) In the skin of the LW^−^ population, the toxin was especially localized in certain parts of Type I cells of the poison glands (I), within the mucous glands (m) and in the blood vessels (v). (**b**) In the SC^+^ population, strong TTX labelling is seen in across the Type I cells of the poison glands. Note that epidermis also shows some labelling of TTX. The insert shows that within the blood vessels TTX labelling is exclusively in the blood plasma. (**c**) High magnification of poison gland of a LW^−^ specimen. (**d**) High magnification of poison gland of a SC^+^ specimen. (**e**) Skin mucous gland of a LW^−^ specimen. Note that only one of the cell types was labelled for TTX. (**f**) Skin mucous gland of a SC^+^ specimen. The cells composing the mucous glands are similarly labelled in both populations. Note that the skin mucous glands in SC^+^ stain less intensely than in the LW^−^ due to the antibody dilution used. Immunolabeling using anti-TTX antibody (1:15000 for LW^−^ and 1:60000 for SC). (**g**) Control of the immunohistochemical method of a LW^−^ specimen. The brownish regions pointed by the arrows indicate pigments that are present in the skin. (**h**) Control of the immunohistochemical method of a skin poison gland from a SC^+^ specimen. For the control, the reaction with peroxidase was also employed, but using 0.2% bovine serum albumin instead of the anti-TTX antibody. Sexes of the animals: male (images **a**–**d**,**h**), female (**e**–**g**).

In the two newt populations, TTX immunolabelling was generally poor for both the epidermis and dermis. However, TTX labelling was much more pronounced and specific in the cutaneous glands. The blood plasma of the dermal capillaries was also marked (Fig. 3a,b). Controls did not show any nonspecific labelling (Fig. 3g,h).

In the poison glands, TTX labelling was stronger in Type I cells, especially in the flocculate granules, but also in the cytoplasm (Fig. 3a–d). Consequently, labelling was much more evident in the SC^+^ population, which presents the most voluminous Type I cells completely filled with flocculate granules, than the LW^−^ population, which possess fewer of these granules within the cells (Fig. 3a–d). In Type II cells, TTX labelling was faint and restricted to the nuclei (Fig. 3a,d). Surprisingly, in both newt populations most cells comprising the mucous glands were also marked for TTX presence (Fig. 3e,f). In LW^−^ population TTX immunolabelling was more evident in mucous glands than in poison glands (Fig. 3a,c,e).

### Biochemistry

**Figure 4:**
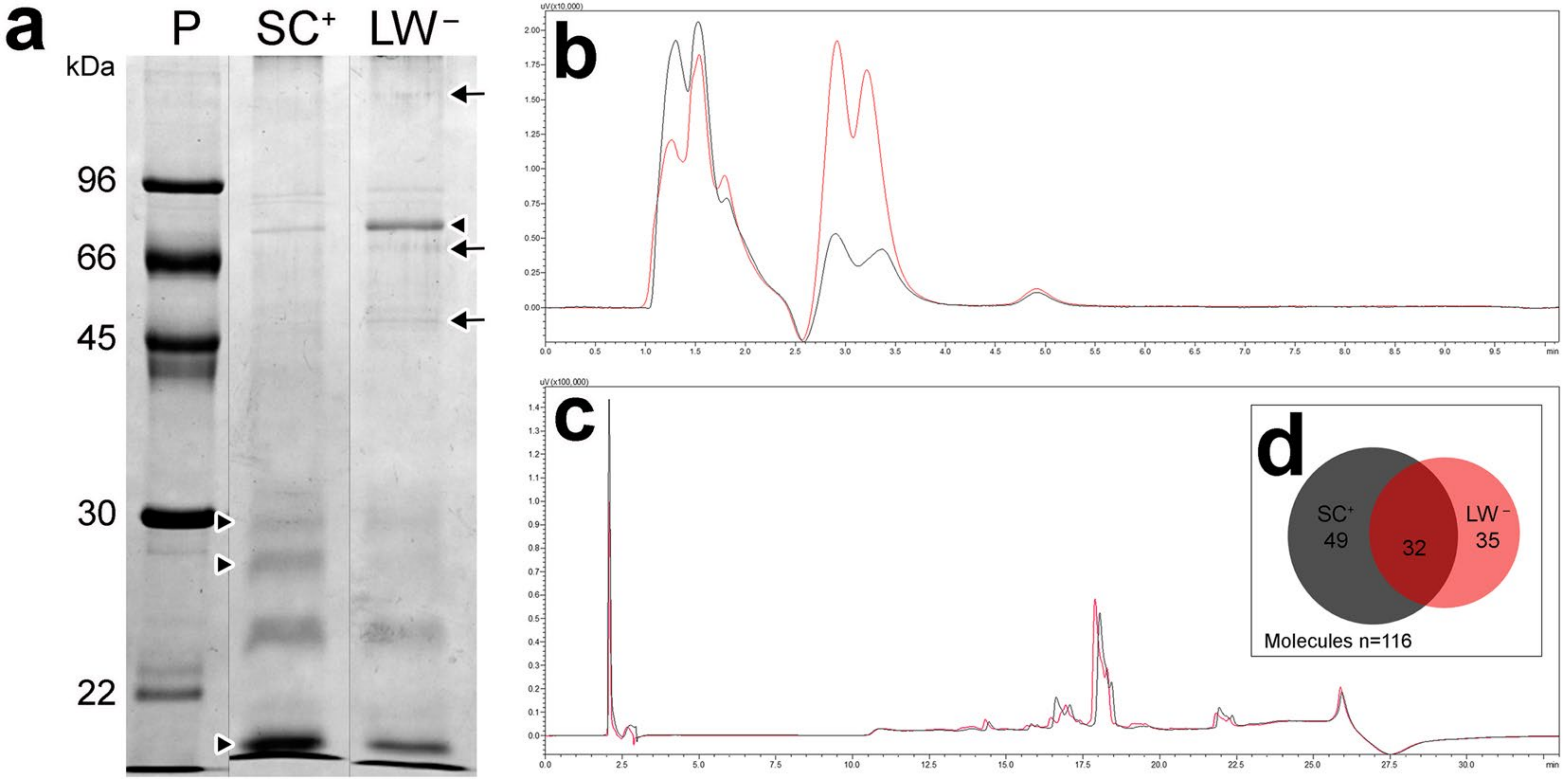
Preliminary characterization of the skin secretion in *Taricha granulosa* from LW^−^ and SC^+^ populations. (**a**) Poison SDS-PAGE profile in both populations (See Fig. S3 for the original image). The arrows indicate the qualitative differences and the arrowheads point to quantitative differences. The numbers at the left (kDa) correspond to the molecular mass markers used as patterns (P). (**b**) C4-RP-HPLC chromatographic profiles obtained from the aqueous extraction of SC^+^ (black) and LW^−^ (red) poisons. (**c**) C8-RP-HPLC chromatographic profiles obtained from the methanolic extraction of SC^+^ (black) and LW^−^ (red) poisons. (**d**) The circle intersection graph shows the sharing of molecules that were found by mass spectrometry. Note the largest number of exclusive molecules in SC^+^ population.

When analyzed by SDS-PAGE, skin secretions obtained from LW^−^ and SC^+^ populations showed similar protein profiles (Fig. 4a), albeit with subtle differences in composition, most notably in LW^−^ population, in which a larger diversity of bands was observed (Figs. 4a and S3). The populations also differed quantitatively, as indicated by the intensity of the bands in the range of 10–30 kDa, but also in a band around 80 kDa (Fig. 4a).

Chromatographic profiles of the skin secretions extracted from the two populations were also very similar for both aqueous and methanolic extraction (Fig. 4b,c respectively). However, the aqueous extraction revealed greater variation in intensity, especially in relation to the second set of peaks eluted between 2.5–3.5 min (Fig. 4b).

Despite the subtle chromatographic differences, mass spectrometry revealed larger differences between the two populations, with the SC^+^ population being richer in low-molecular mass molecules than the LW^−^ population (Fig. 4d and Supplementary Data). Further, the SC^+^ population skin secretion possessed more exclusive molecules, such as the TTX itself (Supplementary Data). Among all detected molecules, only 27% are shared between the two populations (Fig. 4d and Supplementary Data). None of the molecules recorded showed ion fragmentation pattern compatible with TTX analogues.

## Discussion

The two populations of *T. granulosa* did not differ in either the number or total volume of poison glands, suggesting that these variables were not responsible for differences in TTX levels. In contrast, the populations differed with respect to the relative volume of two types of cells within the poison glands. Specifically, we observed a significantly greater volume of Type I cells in the high-TTX SC^+^ population than the low-TTX LW^−^ population, pointing to a relationship between Type I cell volume and amount of TTX. We confirmed this relationship by demonstrating that Type I cells store TTX, specifically in the form of flocculate granules. These flocculate granules represent the majority of granules present in Type I cells in the SC^+^ population but are comparatively rare in the LW^−^ population. Both the volume of Type I cells and the distinct morphology of the poison granules appear to correlate with TTX levels.

Poison glands were more abundant than mucous glands in both studied populations, which is contrary to most previously studied amphibians^21,22^. However, *T. granulosa* poison glands showed a morphology similar to that described in other salamanders, being composed of at least two types of multinucleate cells^19,22–24^ which indicates a syncytial nature^25,26^.

Surprisingly, in addition to the presence of TTX in the poison glands, we also identified TTX within the mucous glands. This result was especially evident in the population with low levels of TTX in which the toxin was more evident in mucous glands than in poison glands. These data are particularly important because they demonstrate the presence of a toxin within the mucous glands, overturning the widely held view that mucous glands are exclusively responsible for mucus secretion^21,22^ and expanding currently held interpretations of amphibian skin gland functionality.

Among amphibians, the morphology of the poison granules is generally related to the maturation process of the skin secretion^25,26^. As a consequence, granules of variable sizes and structures are usually found within the same gland^25^. Based on our results, it seems possible that in *T. granulosa* the dense granules of Type I cells, as observed in LW^−^ population, would undergo a process of maturation. This hypothesis can be tested directly by examining the changes in glandular morphology during the regeneration following toxin depletion.

Our biochemical analysis of the skin secretion highlighted differences in composition between the two populations in relation to low molecular mass compounds and proteins. Such differences must reflect physiological diversity between the two newt populations. Further, it is unclear if the different molecules are involved in chemical defense or are part of the machinery necessary for TTX synthesis or sequestration if TTX is endogenously controlled within *T. granulosa* skin glands. On the other hand, in both populations no molecules with fragmentation pattern similar to TTX analogs were found, a feature that differs from most TTX-bearing amphibians^27,28^.

The origin of TTX in *Taricha granulosa*, as well as in other amphibians, remains uncertain. The origin of TTX in newts may be endogenous, from a symbiont, or result from biological magnification (or some combination of these). The case against symbiosis/sequestration and for endogenous biosynthesis is built from several pieces of evidence: absence of TTX-producing bacteria in/on skin^18^, increasing amounts of TTX over time in captivity fed a controlled (non-TTX containing) diet, and rapid regeneration of TTX following extraction of skin secretion^29^. Definitive evidence is still lacking on the origin of TTX in any amphibian. Thus, at the present it is not possible to support the hypothesis of a relationship between the origin of TTX in *T. granulosa* and the presence of endosymbiotic bacteria within the skin glands, or even other organs, such as the gut, similarly to pufferfish^2^.

Our data corroborate previous findings that TTX is concentrated in poison glands, but also show for the first time that TTX levels are directly related to morphology of skin glands and their secretions. Additionally, we show that the composition of skin secretions from individuals of these two populations is variable. Further investigation is needed to determine whether these differences are simply the result of variation in the amount of TTX that is present or are causally related to TTX production/bioaccumulation.

## Methods

### Animals and TTX quantification

*Taricha granulosa* of both sexes were collected by hand from Soap Creek ponds (SC^+^; Corvallis, OR; body weight mean 11.84 g ± 1.92 g standard deviation, mean total length 154.88 mm ± 14.86 mm standard deviation) and from Lake in the Woods (LW^−^; Glide, OR; 10.4 g ± 2.37 g, 168.33 mm ± 19.36 mm), USA in May 2015. Newts from these populations are known to possess high^10^ and low^30^ TTX levels, respectively. To relate the origin of the newts to the TTX levels, we used the acronym SC^+^ for the population with high levels of TTX and LW^−^ to discriminate the population with low levels of TTX. The animals were transported to Utah State University where they were housed individually in plastic containers with 2 L filtered tap water in an environmental chamber at 6 °C. Animals were kept under a 14:10 h light day cycle and fed twice a week with blackworms (*Lumbriculus variegatus*), a TTX free organism^31^. Dorsal skin was sampled from four, randomly chosen specimens of each population using a 3 mm biopsy punch to quantify TTX levels using a Competitive Inhibition Enzymatic Immunoassay^32^. Whole skin TTX amount was estimated for each newt using the method of Hanifin^33^, which is a predictive model based on measures of the amount of TTX present in the dorsal skin. All procedures were approved by the Utah State University Institutional Animal Care and Use Committee (# 2157) and all methods were performed in accordance with the relevant guidelines and regulations.

### Histology

Dorsal skin was sampled from four, randomly chosen specimens of each population using a 3 mm biopsy punch. These samples were fixed in buffered formalin for 24 h, dehydrated in alcohol, embedded in paraffin or historesin, sectioned, and stained with hematoxylin-eosin or toluidine blue-fuchsine for morphological analysis. For chemical composition characterization, the sections were submitted to the following histochemical methods^34^: bromophenol blue (for proteins) and alcian blue pH 2.5 and PAS (for acid and basic mucopolysaccharides, respectively).

### Poison gland quantification

To determine the number of poison glands, dorsal skin was sampled from four, randomly chosen specimens of each population using a 3 mm biopsy punch, preserved in formalin, and then stained with 0.1% Coommassie Blue R250 solution in 10% acetic acid, 50% methanol, and 40% water for 1 h. The punches were decolorized in a solution of 10% acetic acid, 50% methanol, and 40% water for 4 days and transferred to pure glycerol. Each punch was placed between two histological slides and viewed under a light microscope using the 4x objective. With the aid of the LAS software (LAS V4.6, Leica Microsystems; Wetzlar, Germany), the number of poison glands in an area of 1 mm^2^ of skin was counted.

### Tridimensional reconstruction and morphometrics of poison glands

Tridimensional reconstructions of poison glands were based in 6 µm paraffin serial sections of randomly chosen whole glands using the software Reconstruct^35^. The volume of the secretory cells of one poison gland chosen randomly from each specimen was calculated by multiplying the area (µm^2^) occupied in each section by the section thickness. Statistical comparison of morphometric data from SC^+^ and LW^−^ populations was carried out using Student’s *t*-test. Differences were considered statistically significant when p ≤ 0.05.

### Scanning electron microscopy

Skin samples of two randomly chosen specimens of each population were fixed following the same protocol described previously, immersed in dimethylsulfoxide (DMSO), frozen in liquid nitrogen, and fractured with the aid of a frozen steel blade^26^. After dehydration in ethanol, the samples were mounted on aluminum stubs, dried in a critical point apparatus, covered with gold in a sputtering device, and examined in a FEI Quanta 250 scanning electron microscope, operating at 10–12 kV.

### Immunohistochemistry

The technique employed was adapted from Mebs^20^. Paraffin sections from four randomly chosen specimens of each population were treated with 0.3% hydrogen peroxide and 20% bovine serum albumin and incubated with commercial monoclonal anti-TTX antibody (1:15000 for LW^−^ and 1:60000 for SC^+^; Hawaii Biotech) followed by biotin-conjugated anti-mouse IgG (Dako®). As an enzyme marker, the avidin and biotinylated horse radish peroxidase macromolecular complex kit (Dako®) was used, and distribution of the antigen in skin was visualized by 3,3′-diaminobenzidine (DAB) substrate solution for 30 sec. Sections were counterstained with hematoxylin. Positive stain of the antigen was recognized as a goldish or brownish color. For controls, we employed the same method described above using 0.2% bovine serum albumin instead of the anti-TTX antibody.

### Skin secretion collection

Glandular secretions were extracted from the dorsum of 12 randomly selected live newts of each population with the use of a bipolar stimulating electrode (Astro-med, Inc.). Secretions of specimens of each population were pooled, frozen, lyophilized, and stored at −20 °C until biochemical characterization.

### Electrophoresis (SDS-PAGE)

In order to analyze the protein profiles, skin secretion samples of SC^+^ and LW^−^ newts were loaded onto a 12% polyacrylamide gel (PAGE) containing sodium dodecyl sulphate (SDS) under reducing conditions^36^. After separation of the proteins by electrophoresis, the gels were stained with Coomassie brilliant blue.

### RP-HPLC and mass spectrometry

The secretions were processed in order to obtain two extracts and remove molecules with different hydrophobicity. Ultrapure water was first added to the secretion; the solution was then homogenized and centrifuged for 5 min at 5000 × g. The supernatant was separated (termed water extract) and methanol was added to the pellet, which was solubilized and centrifuged in the same way as the water extract (termed methanol extract). Both extracts were cleaned using ZipTip® and the eluted content was analyzed by HPLC and mass spectrometry.

The water extract was injected in a C4 column (2.1 mm × 50 mm, 5 µm) coupled to an HPLC system (Proeminence 20 A, Shimadzu Co., Japan) and the elution was performed by isocratic method with a 20 mM triethylamine, 20 mM ammonium formate, and 1% acetonitrile buffer (pH 4.5). The flow was set constant at 0.2 mL/min and molecules were directly introduced in the mass spectrometry (ESI-IT-ToF, Shimadzu Co., Japan), operating in positive ionization mode and full scan, ranging from 50–2000 m/z. The interface voltage was kept at 4.5 kV, the detector voltage at 1.76 kV, and the capillary temperature at 200 °C. The instrument control and data acquisition were conducted using LC Solutions (Shimadzu Co., Japan).

The methanol extract was also analyzed using HPLC/mass spectrometry, using a C8 column (ACE, 2.1 mm × 100 mm, 5 µm) and elution by a two solvent system (A = ultrapure water containing acetic acid 0.1% and B = 90% acetonitrile in ultrapure water, containing acetic acid 0.1% containing acetic acid 0.1%). The column was eluted by a gradient of 0–100% B in 20 min. The flow was set constant at 0.2 mL/min and the parameters of mass spectrometry were the same as mentioned above.

For TTX identification, the 320.1 m/z ion was monitored in the aqueous sample and the ion was fragmented by argon gas, with collision energy kept in 50%, in order to confirm the daughter ion profile and, consequently, the identity of TTX. The presence of TTX analogues was investigated by the search of ion fragments 162 m/z and 178 m/z^37^.

## Supplementary information

Supplementary

## Data Availability

All data produced in this study are included in the text and supplementary information documents.
