# Supplementary material for: Variations in tetrodotoxin levels in populations of Taricha granulosa are expressed in the morphology of their cutaneous glands
Source: Sci Rep. 2019 Dec 6;9:18490. doi: 10.1038/s41598-019-54765-z (PMC6897900; doi:10.1038/s41598-019-54765-z)
Supplement: Supplementary file 1 — Supplementary [file 41598_2019_54765_MOESM1_ESM.docx]

**Supplementary information for: “Variations in tetrodotoxin levels in populations of *Taricha granulosa* are expressed in the morphology of their cutaneous glands”**

Pedro Luiz Mailho-Fontana*, Carlos Jared, Marta Maria Antoniazzi, Juliana Mozer Sciani, Daniel Carvalho Pimenta, Amber N. Stokes, Taran Grant, Edmund D. Brodie III & Edmund D. Brodie Jr.

*Correspondence to: Pedro Luiz Mailho-Fontana (mailho.fontana@gmail.com)

**Supplementary Table S1.** **TTX concentration in** **Lake in the Woods (LW^–^) and Soap Creek (SC^+^) newts**. TTX amounts in whole skin of each newt was estimated using the method of Hanifin^32^.

| Samples | Sex | Final concentration (ng/mL) | TTX/cm^2^ (ng) | Newt Mass (g) | Skin area (cm^2^) | TTX amounts in whole skin of each newt (mg) |
| --- | --- | --- | --- | --- | --- | --- |
| LW^–^ | Female | 0.44 | 6.2673 | 12.2 | 53.22 | 0.000173144 |
| LW^–^ | Female | 0 | 0 | 16.6 | 66.41 | 0 |
| LW^–^ | Female | 0 | 0 | 12.2 | 53.22 | 0 |
| LW^–^ | Male | 0 | 0 | 11.3 | 50.37 | 0 |
| SC^+^ | Female | 2127.10 | 30098.4759 | 16.8 | 66.99 | 1.046659721 |
| SC^+^ | Male | 2520.54 | 35665.6123 | 16.3 | 65.55 | 1.213593968 |
| SC^+^ | Male | 2289.95 | 32402.8356 | 16.5 | 66.13 | 1.11232723 |
| SC^+^ | Female | 2604.27 | 36850.4598 | 18.6 | 72.07 | 1.37863234 |

**Figure S1. Histochemical characterization of the cutaneous glands of *Taricha granulosa* from the LW^–^ and SC^+^ populations.** (a) In the SC^+^ population, only Type II cell granules show protein content. (b) In the LW^–^ population, in addition to the protein content in Type II cell granules, the dense granules of Type I cells also contain protein. The insert shows labeling of the dense granules, indicating protein content. In the SC^+^ population (c) and in the LW^–^ population (d), the poison glands did not show neutral mucopolysaccharides. These compounds were found only within one type of cell present in the mucous glands. In the SC^+^ population (e) and in the LW^–^ population (f) both poison and mucous glands are negative for neutral mucopolysaccharides. Methods: bromofenol blue (a and b), PAS (c and d) and alcian blue pH 2,5 (d and f). Sexes of the animals: female (images a-f).


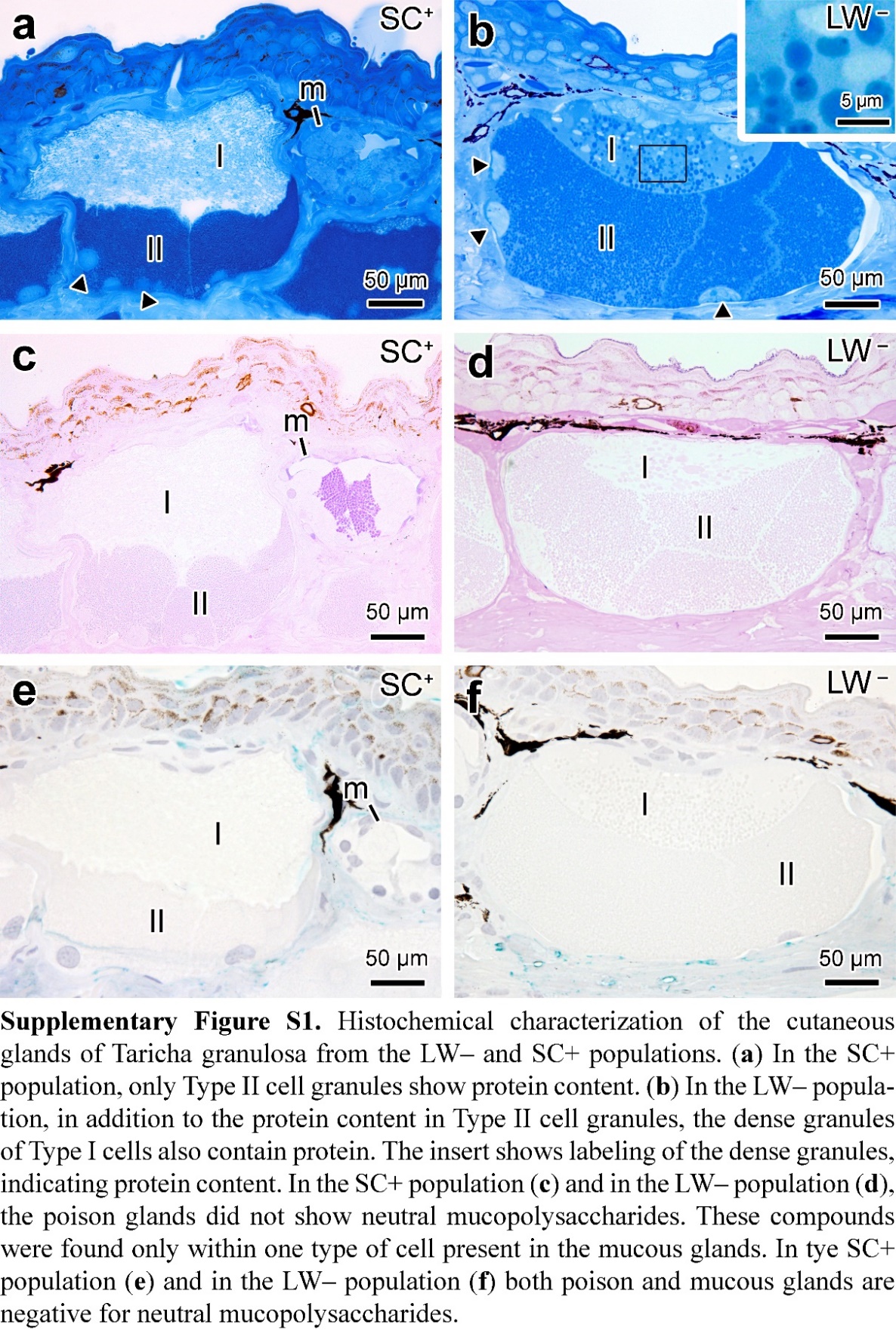


**Figure S2. Original image of the gel used in Fig. 4.** Molecular mass markers used as patterns (P)**,** Soap Creek (SC^+^) and Lake in the Woods (LW^–^) skin secretion.


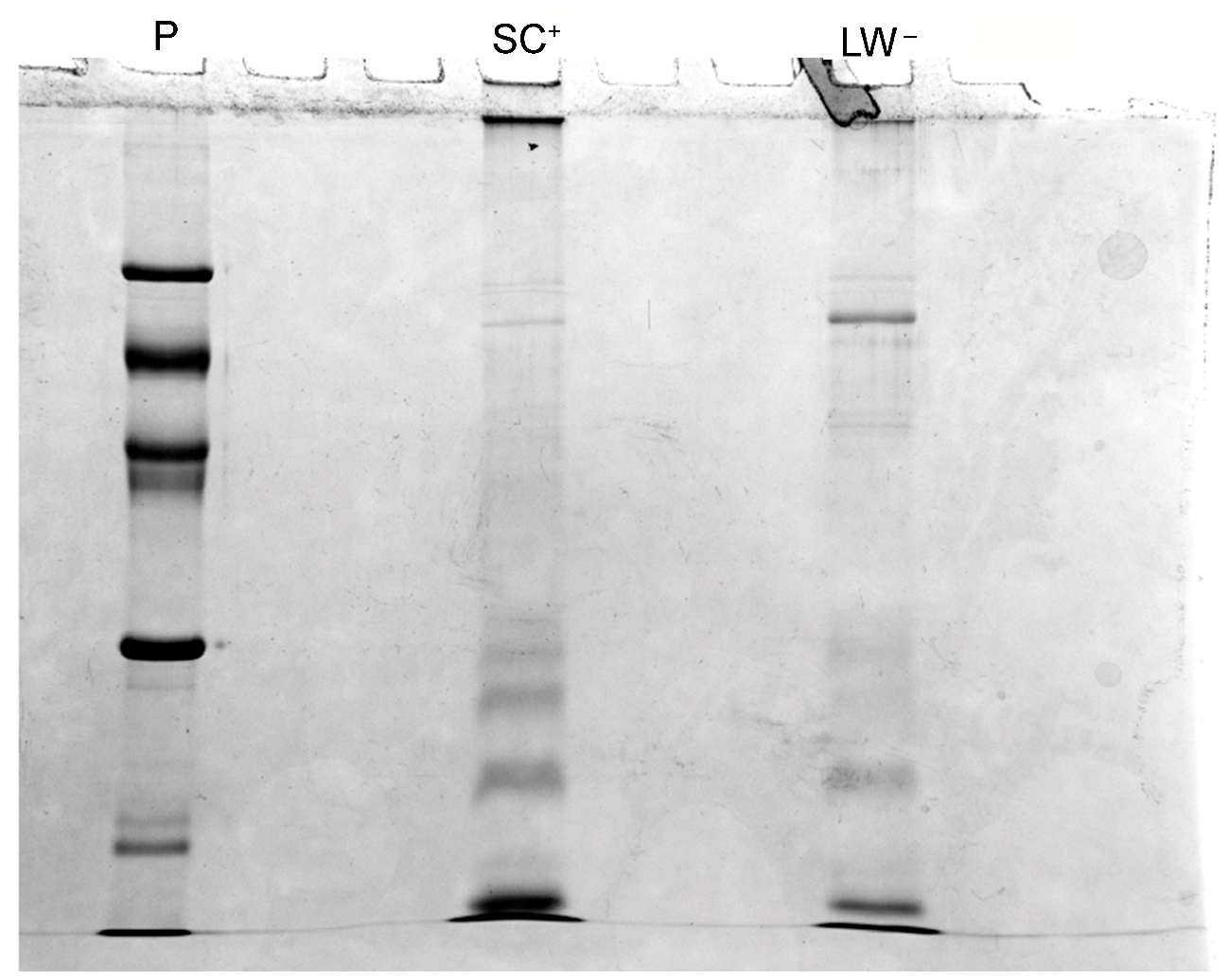


**Supplementary Data.** **Mass ratio (m/z) of the chemical compounds identified in secretions of Lake in the Woods (LW–) and Soap Creek (SC+) newt populations.** “X” represents the presence of m/z. Asterisk (*) indicates tetrodotoxin.

| m/z | *Taricha granulosa* | |
| --- | --- | --- |
|  | LW^–^ | SC^+^ |
| 104.1 | X |  |
| 154.48 | X |  |
| 238.12 |  | X |
| 238.69 |  | X |
| 238.97 | X | X |
| 240.05 | X | X |
| 240.21 | X | X |
| 240.23 | X | X |
| 240.25 |  | X |
| 241.05 |  | X |
| 241.1 | X | X |
| 241.12 |  | X |
| 241.16 |  | X |
| 241.22 | X | X |
| 241.36 | X | X |
| 241.49 | X | X |
| 241.5 |  | X |
| 242.22 | X |  |
| 270.23 |  | X |
| 279.18 | X |  |
| 299.18 | X |  |
| 302.3 |  | X |
| 316.73 | X |  |
| 316.89 | X | X |
| 316.91 | X |  |
| 316.97 | X |  |
| 318.01 | X | X |
| 318.06 |  | X |
| 318.14 | X | X |
| 318.22 | X | X |
| 318.23 | X | X |
| 318.26 |  | X |
| 318.28 | X | X |
| 318.29 | X | X |
| 318.31 | X | X |
| 320.09* |  | X |
| 331.25 |  | X |
| 338.32 | X | X |
| 338.38 |  | X |
| 358.35 | X | X |
| 358.4 | X |  |
| 359.28 | X | X |
| 365.03 | X | X |
| 367.05 |  | X |
| 367.12 |  | X |
| 368.27 | X |  |
| 380.32 | X |  |
| 381.41 | X |  |
| 399.27 |  | X |
| 402.03 | X | X |
| 402.35 | X |  |
| 404.05 | X |  |
| 438.46 |  | X |
| 441.14 |  | X |
| 443.04 |  | X |
| 444.95 | X |  |
| 448.69 | X |  |
| 456.44 |  | X |
| 476.24 |  | X |
| 494.59 |  | X |
| 499.44 | X |  |
| 504.21 | X | X |
| 512.14 |  | X |
| 512.48 | X | X |
| 521.12 |  | X |
| 531.38 |  | X |
| 536.03 | X | X |
| 536.14 | X | X |
| 536.16 | X | X |
| 536.22 | X |  |
| 538.05 | X |  |
| 538.15 | X |  |
| 540.59 | X |  |
| 542.2 |  | X |
| 550.55 | X | X |
| 550.69 | X |  |
| 599.09 |  | X |
| 599.97 | X |  |
| 600.45 |  | X |
| 600.46 | X |  |
| 610.14 | X | X |
| 610.2 | X | X |
| 612.06 | X | X |
| 628.08 |  | X |
| 632.08 |  | X |
| 645.34 | X |  |
| 648.02 |  | X |
| 658.48 |  | X |
| 684.1 |  | X |
| 684.25 |  | X |
| 685.19 | X |  |
| 704.84 | X |  |
| 705.01 |  | X |
| 712.13 |  | X |
| 724.36 | X |  |
| 726.88 |  | X |
| 764.51 | X | X |
| 764.55 | X | X |
| 765.57 |  | X |
| 768.97 |  | X |
| 803.22 |  | X |
| 844.4 |  | X |
| 868.07 |  | X |
| 883.9 | X |  |
| 933.86 | X |  |
| 968 |  | X |
| 989.89 |  | X |
| 998.72 | X |  |
| 1024.8 |  | X |
| 1044.3 | X |  |
| 1071.3 |  | X |
| 1168.5 | X |  |
| 1184.7 |  | X |
| 1193.4 |  | X |
| 1203.5 | X |  |
| 1327.9 | X |  |
